# Nomophobia among university students in five Arab countries in the Middle East: prevalence and risk factors

**DOI:** 10.1186/s12888-023-05049-4

**Published:** 2023-07-26

**Authors:** Abdallah Y Naser, Hassan Alwafi, Rania Itani, Salman Alzayani, Sami Qadus, Rabaa Al-Rousan, Ghada Mohammad Abdelwahab, Eman Dahmash, Ahmad AlQatawneh, Hani M J Khojah, Angga Prawira Kautsar, Renan Alabbasi, Nouf Alsahaf, Razan Qutub, Hamzeh Mohammad Alrawashdeh, Amer Hamad Issa Abukhalaf, Mohamed Bahlol

**Affiliations:** 1 Department of Applied Pharmaceutical Sciences and Clinical Pharmacy, Faculty of Pharmacy, Isra University, Amman, Jordan; 2 Faculty of Medicine, Umm Al Qura University, Mecca, Saudi Arabia; 3 Pharmacy Practice Department, Faculty of Pharmacy, Beirut Arab University, Riad El Solh, Beirut, Lebanon; 4 Department of Family and Community Medicine, Arabian Gulf University, Manama, Bahrain; 5 Department of Chemical and Pharmaceutical Sciences, School of Life Sciences, Pharmacy and Chemistry, Faculty of Health, Science, Social Care and Education, Kingston University, London, UK; 6 ACDIMA Center for Bioequivalence and Pharmaceutical Studies, Department of Clinical Research, Amman, Jordan; 7 Department of Clinical and Hospital Pharmacy, College of Pharmacy, Taibah University, Madinah, Kingdom of Saudi Arabia; 8 Unit of Global Health, Department of Health Sciences, University of Groningen/University Medical Center Groningen, Groningen, the Netherlands; 9 Department of Pharmaceutics and Pharmaceutical Technology, Faculty of Pharmacy, Universitas Padjadjaran, Bandung, West Java Indonesia; 10 Center of Excellence in Higher Education for Pharmaceutical Care Innovation, Universitas Padjadjaran, Bandung, West Java Indonesia; 11 Department of Ophthalmology, Sharif Eye Centers, Irbid, Jordan; 12 Florida Institute for Built Environment Resilience, M.E. Rinker, Sr. School of Construction Management, College of design construction and planning, University of Florida, Gainesville, FL USA; 13 Specialty of Pharmaceutical Management and Economics, Department of Pharmacy Practice and Clinical Pharmacy, Egyptian Russian University, Cairo, Egypt

**Keywords:** Bahrain, Egypt, Jordan, Lebanon, Nomophobia, Saudi Arabia, University students

## Abstract

**Background:**

Excessive use of mobile phones leading to development of symptoms suggestive of dependence syndrome with teenagers are far more likely to become dependent on mobile phones as compared to adults. COVID-19 pandemic has had an impact on the mental health of several groups in society, especially university students. This study aimed to explore the prevalence of mobile phone dependence among university students and its associated factors.

**Methods:**

Between September 2021 and January 2022, a cross-sectional study was conducted at universities in Jordan, Lebanon, Egypt, Bahrain, and Saudi Arabia utilizing an online and paper-based self-administered questionnaire. We employed a previously developed questionnaire by Aggarwal et al.

**Results:**

A total of 5,720 university students were involved in this study (Egypt = 2813, Saudi Arabia = 1509, Jordan = 766, Lebanon = 432, and Bahrain = 200). The mean estimated daily time spent on using mobile phone was 186.4 (94.4) minutes. The highest mobile dependence score was observed for the university students from Egypt and the lowest mobile dependence score was observed for the university students from Lebanon. The most common dependence criteria across the study sample was impaired control (55.6%) and the least common one was harmful use (25.1%). Females and those reported having anxiety problem or using a treatment for anxiety were at higher risk of developing mobile phone dependence by 15% and 75%, respectively.

**Conclusion:**

Mobile phone dependence is common among university students in Arab countries in the Middle East region. Future studies exploring useful interventions to decrease mobile phone dependence are warranted.

## Background

The introduction of the mobile phone (cell phone, smartphone, and feature phone) [1] is one of the most significant technological developments of the past three decades [1]. Mobile phone has many attributes and characteristics that make it very attractive particularly to adolescence [2], yet they have become an integral part of the lives of men and women of all ages and play a significant role in causing behavioral addiction [3]. With the continuous development of mobile capabilities, the use of mobile phones continues to increase in all areas, including situations where concentration is vital, such as while driving. According to the United States Department of Transportation, in 2020, approximately 8.1% of all traffic fatalities were attributable to cell phone distracted driving. In addition, 325,000 individuals were injured in distracted driving accidents. Drivers between the ages of 20 and 30 had the highest rate of distracted driving-related fatalities, accounting for 7% of all fatalities [5, 6]. In 2022, there were 7.26 billion mobile phone subscribers worldwide, representing 91.7% of the global population. Out of this figure, the number of smartphone users was 6.648 billion (84.0%) [7] [8, 9].

The terms ‘‘mobile phone problematic use’’ (MPPU), ‘‘problem cell phone use’’(PCPU), “mobile phone dependence (MPD)”, and ‘‘mobile phone abuse or addiction’’ have been used to describe patterns of interaction with a mobile phone that have the characteristics of addiction [10]. MPPU is a form of ‘‘cyber-disorder,’’ cyber addiction, or a behavioral (technological) addiction, and is characterized by repetitive use of the mobile phone to engage in behavior that is known to be counterproductive to health [11]. Scientifically, such behavioural mobile phone addiction is characterized as “mobile phone mania,” a state of socio-psychological illness, and it is clinically referred as “Nomophobia” which is a portmanteau for (“NO MObile PHOne” and “phoBIA”) [12].

Nomophobia is defined as the anxiety of being without a mobile phone, and nomophobes are those who exhibit an addiction to their mobile phone [2]. The Diagnostic and Statistical Manual of Mental Disorders, Fourth Edition (DSM-IV) classified nomophobia as a “phobia for a particular/specific thing” [13] [14].Literature indicates that excessive use of mobile phones leads to the development of symptoms suggestive of dependence syndrome, with adolescents being far more likely than adults to become dependent on mobile phones [15–17]. In a study conducted in 2015 on 415 Indian students, Nikhita, CS. et al. reported that MPD was found in 31.1% of the surveyed samples and it was significant for gender, phone type, average use per day and years of using the device [12].

There has been a considerable number of research studies that investigated mobile phone addiction and its associated risks to health [3, 18]. Studies were conducted in Thailand [19], Korea [8], India [12, 15], the UK [10], Japan [20], Spain [21], Turkey [22], Australia [23], the Philippines [24], and in Lebanon [25]. All these studies found a significant positive correlation between mobile phone utilization patterns and the severity of nomophobia [26].

Mental, physical, and psychological health are impacted by excessive mobile phone dependence [27][11]. Individuals with nomophobia [28]when they lose their mobile phone or run out of battery and network, they become terrified, irritated, and maybe psychologically abnormal [29]. Behavioral addictions have been understood as equivalent to substance dependence or as more analogous to the obsessive-compulsive spectrum and thus, some researchers deem that it has become important to consider MPD as a diagnostic entity [1].

The above findings give indication how serious and widespread this “psychological dependence” or addiction is and the need to conduct research in our region of the world to find out the magnitude of this issue compared to rest of the world and based on those findings, to propose appropriate guidelines to make people aware of the negative consequences of their mobile phones. For this reason, a survey study was conducted on university students from five Arabic countries namely Jordan, Saudi Arabia, Lebanon, Egypt, and Bahrain. This study aimed to explore the prevalence of mobile phone dependence among university students and its associated factors.

## Methods

### Study design and study population

Between September 2021 and January 2022, a cross-sectional study was conducted at universities in Jordan, Lebanon, Egypt, Bahrain, and Saudi Arabia utilizing an online and paper-based self-administered questionnaire. Participants in the study were university students from any field of study and at any level who were willing to participate. Participation was entirely voluntary. The study participants’ personal information was not collected. The questionnaire tool gathered demographic information and asked participants about their mobile phone usage patterns.

### Sampling strategy

The study participants were chosen from a convenience sample of eligible people. Participants were contacted by social media (Facebook, Twitter, Snapchat, and Instagram) and a paper-based questionnaire to participate in this study (if feasible). A survey link was used to invite the study sample. All study participants gave their informed consent for inclusion before they participated in the study. The survey’s goals and objectives were stated explicitly at the start of the invitation letter. University students aged 18 years and over who resided in one of the participating countries were eligible to participate. Any participant who did not meet the inclusion criteria was excluded from the study. To enhance response and make the survey accessible to the general public and healthcare professionals, the survey URL was re-posted once a week. Submissions were allowed only allowed after all questions of the online questionnaire have been answered.

### Questionnaire tool

To achieve the study’s goals, we employed a previously developed questionnaire by Aggarwal et al. [1]. The original questionnaire consisted of 20 questions that were aimed to offer information on mobile usage patterns and whether or not such patterns met the ICD-10 criteria for substance dependence syndrome. The first three questions focused on the length of time spent on mobile phones in years, the average amount of time spent on mobile phones each day, and the purpose of use. The remaining 20 items were a questionnaire with a binominal (yes/no) response that asked about mobile usage patterns and if they met the ICD-10 criteria for dependent syndrome. A total of 14 of the 20 items addressed the six ICD-10 dependency syndrome criteria (one question for intense desire, four questions for impaired control, three questions for withdrawal, one question for tolerance, four questions for decreased pleasure, and one question for harmful use). Participants were judged to have met a criterion if they answered yes to all questions in single-question criteria or yes to at least half of the questions in multiple-question criteria [1]. Participants were classified as having mobile phone dependence if they met three or more of the ICD-10 criteria for dependence. Students’ mobile dependence scores were obtained by assigning a one-point score to each positive (yes) response to the 20-items that explored mobile dependence. The higher the score, the more mobile-dependent the student is. The forward-backward technique was utilized to translate the questionnaire into Arabic, which was then employed in this study.

### Sample size

The required sample size from each study population was 385 participants, based on a confidence interval of 95%, a standard deviation of 0.5, and a margin of error of 5%.

### Statistical analysis

IBM Corp.‘s Statistical Package for Social Science (SPSS) software, version 27 (IBM Corp, Armonk, NY, USA), was used to analyze the data. Categorical variables were reported as frequencies and percentages. For continuous variables, the descriptive analysis was reported as mean (standard deviation [SD]).t-test and one-way ANOVA were used to compare the mean scores for mobile phone dependency between different demographic groups. A Fisher’s least significant difference (LSD) post-hoc test was conducted to identify the source of significant variation within each group. Binary logistic regression was conducted to determine factors associated with having nomophobia symptoms. The cut-off point for the logistic regression was fulfilling three or more of the ICD-10 criteria for substance dependence syndrome. A confidence interval of 95% (P < 0.05) was applied to represent the statistical significance of the results, and the level of significance was predetermined as 5%.

## Results

A total of 5,720 university students were involved in this study from five Middle Eastern countries (Egypt = 2813, Saudi Arabia = 1509, Jordan = 766, Lebanon = 432, and Bahrain = 200). The vast majority of them (92.5%) were aged below 29 years. More than half of them (66.1%) were females. Around 90.0% were single. More than half of them (55.9%) were studying at medical schools. Around 80.0% of them reported that their monthly income category is below 700$. Around one-third (29.9%) the study participants reported that they suffer from an anxiety problem or use a treatment for anxiety. The mean estimated daily time spent on using mobile phone was 186.4 (94.4) minutes. Around 43.0% of the study participants reported that they are using mobile phones since 6–10 years. For further details on the sociodemographic characteristics of the study participants, refer to Table 1.

**Table 1:** Participants demographic characteristics

Demographic variable	Overall (n = 5720)		Egypt (n = 2813)		Saudi Arabia (n = 1509)		Jordan (n = 766)		Lebanon (n = 432)		Bahrain (n = 200)	
	Frequency	Percentage	Frequency	Percentage	Frequency	Percentage	Frequency	Percentage	Frequency	Percentage	Frequency	Percentage
**Age categories** (years) (n = 5715)												
18–29 years	5290	92.5%	2785	99.2%	1303	86.3%	663	86.6%	383	88.7%	156	78.0%
30–49 years	342	6.0%	16	0.6%	168	11.1%	98	12.8%	39	9.0%	21	10.5%
50 years and above	83	1.5%	7	0.2%	38	2.5%	5	0.7%	10	2.3%	23	11.5%
**Gender** (n = 5712)												
Females	3773	66.1%	1841	65.6%	971	64.3%	494	64.5%	317	73.4%	150	75.0%
**Marital status** (n = 5716)												
Single	5139	89.9%	2683	95.5%	1213	80.4%	654	85.4%	409	94.7%	180	90.0%
Married	503	8.8%	120	4.3%	247	16.4%	97	12.7%	22	5.1%	17	8.5%
Divorced	58	1.0%	4	0.1%	37	2.5%	13	1.7%	1	0.2%	3	1.5%
Widowed	16	0.3%	2	0.1%	12	0.8%	2	0.3%	0	0.0%	0	0.0%
**Field of study** (n = 5707)												
Medical college	3191	55.9%	1248	44.6%	797	52.8%	616	80.4%	350	81.0%	180	90.0%
**Year level** (n = 5701)												
First year	716	12.6%	224	8.0%	182	12.1%	142	18.5%	89	20.6%	79	39.5%
Second year	772	13.5%	359	12.8%	162	10.7%	152	19.8%	76	17.6%	23	11.5%
Third year	892	15.6%	429	15.4%	240	15.9%	124	16.2%	78	18.1%	21	10.5%
Fourth year	1026	18.0%	577	20.7%	248	16.4%	123	16.1%	68	15.7%	10	5.0%
Fifth year	1443	25.3%	885	31.7%	299	19.8%	163	21.3%	81	18.8%	15	7.5%
Sixth year	281	4.9%	44	1.6%	197	13.1%	7	0.9%	5	1.2%	28	14.0%
Higher education	571	10.0%	276	9.9%	181	12.0%	55	7.2%	35	8.1%	24	12.0%
**Income level** (n = 5504)												
Less than 700$	4415	80.2%	2494	96.0%	990	65.6%	510	66.6%	334	77.3%	87	43.5%
700–1500$	542	9.8%	68	2.6%	177	11.7%	183	23.9%	56	13.0%	58	29.0%
1500–2100$	227	4.1%	12	0.5%	125	8.3%	45	5.9%	23	5.3%	22	11.0%
2100$ and above	320	5.8%	23	0.9%	217	14.4%	28	3.7%	19	4.4%	33	16.5%
**In general, do you suffer from an anxiety problem or use a treatment for anxiety** (n = 5646)**?**												
Yes	1688	29.9%	670	23.9%	524	35.6%	248	33.3%	170	39.4%	76	38.0%
**Mean daily time spent on using mobile phone (SD)** (minutes):												
	186.4 (94.4)		159.7 (102.0)		217.9 (77.6)		198.3 (81.0)		208.9 (74.6)		225.7 (77.3)	
**Duration of mobile phone usage** (years) (n = 5705):												
Less than one year	95	1.7%	57	2.0%	16	1.1%	16	2.1%	2	0.5%	4	2.0%
1–5 years	1200	21.0%	714	25.5%	185	12.3%	205	26.8%	71	16.4%	25	12.5%
6–10 years	2460	43.1%	1144	40.9%	694	46.0%	287	37.5%	250	57.9%	85	42.5%
10 years and above	1950	34.2%	883	31.6%	614	40.7%	258	33.7%	109	25.2%	86	43.0%

### Mobile phone use pattern

**Fig. 1:**
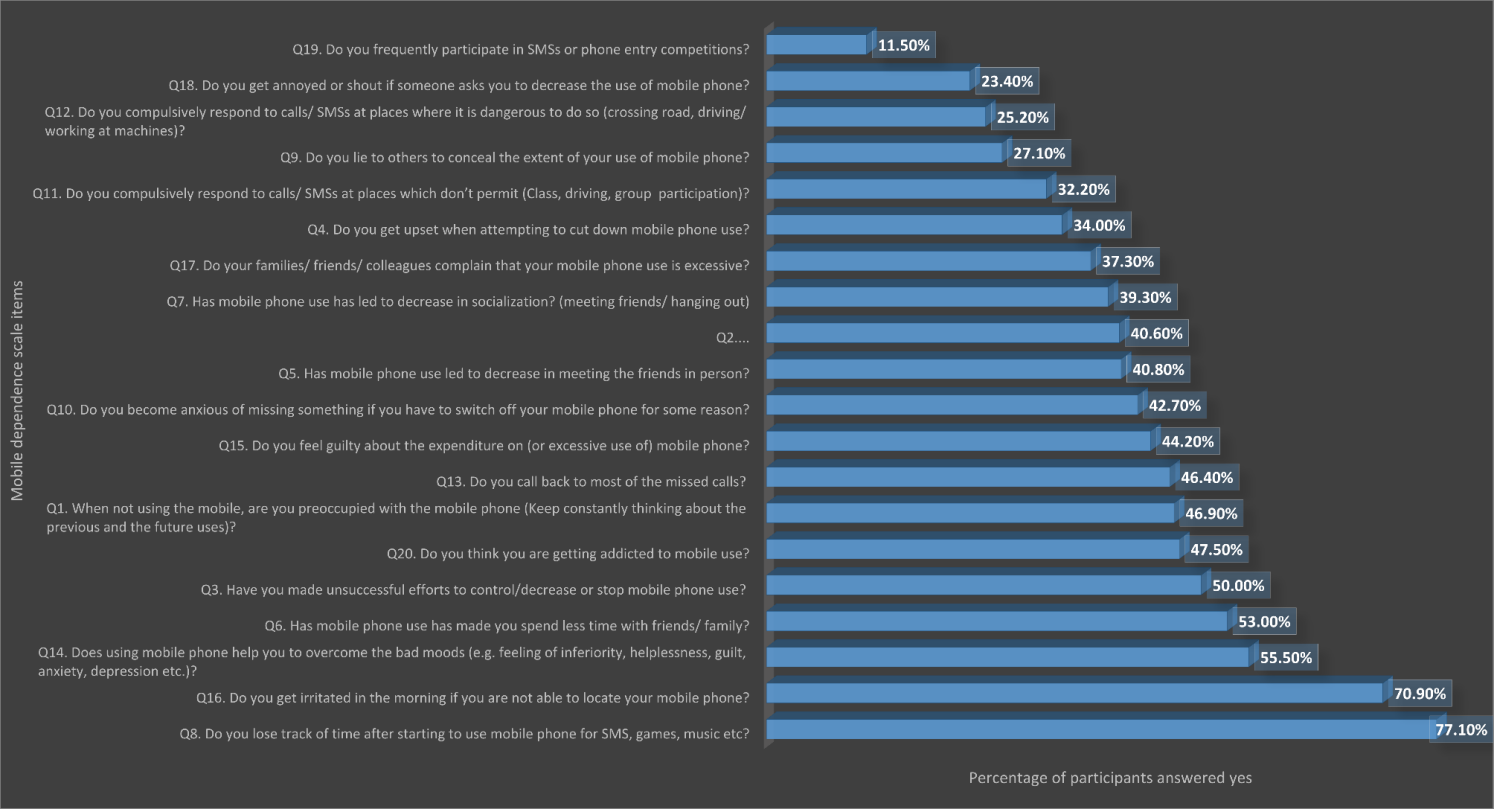
Students response to mobile dependence scale items. SMS: short-message-service

Figure 1 below describes students’ responses regarding their mobile use pattern. Most of the students confirmed that using mobile phone help them to overcome the bad moods (e.g. feeling of inferiority, helplessness, guilt, anxiety, depression etc.), they get irritated in the morning if they are not able to locate their mobile phone, and they get annoyed or shout if someone asks them to decrease the use of mobile phone.

### Students mobile dependence score

The mean mobile dependence score for the study sample was 8.43 (4.41). The highest mobile dependence score was observed for the university students from Egypt (8.62 (4.40)) and the lowest mobile dependence score was observed for the university students from Lebanon (7.43 (4.06), Table 2.

**Table 2:** Mean mobile dependence score stratified by country

Country	Mean mobile phone dependence score (SD)	P-value
Egypt	8.62 (4.40)	p < 0.001
Saudi Arabia	8.52 (4.55)	
Jordan	8.25 (4.35)	
Bahrain	7.97 (3.94)	
Lebanon	7.43 (4.06)	

Table 3 below presents the percentage of university students who fulfil ICD-10 criteria for mobile dependence syndrome. Around one quarter (24.2%) the participating university students are diagnosed (symptomatically according to ICD-10 criteria for dependence syndrome) with mobile phone dependence. The most common dependence criteria across the study sample was impaired control (55.6%) and the least common one was harmful use (25.1%).

**Table 3:** ICD-10 diagnostic criteria for mobile dependence syndrome

ICD-10 Criteria for Dependence syndrome	Percentage of participants
Intense Desire (Q1)	(2677/5720) 46.8%
Impaired control (Q3,8,11,19)	(3295/5720) 57.6%
Withdrawal (Q10,13,16)	(3180/5720) 55.6%
Tolerance (Q2)	(2317/5720) 40.5%
Decreased pleasure (Q5,6,7,17)	(2826/5720) 49.4%
Harmful use (Q12)	(1436/5720) 25.1%
Dependence syndrome among mobile phone users	(1384/5720) **24.2%**

Table 4 presents mobile phone dependence score stratified by students’ sociodemographic characteristics. Mobile phone dependence score differed significantly based on age, gender, field of study, whether the student suffer from an anxiety problem or use a treatment for anxiety, and duration of mobile phone usage.

**Table 4:** Participants mean mobile phone dependence score stratified by their demographic characteristics

**Demographic variable**	**Overall** (n = 5720)		p-value	Egypt (n = 2813)		Saudi Arabia (n = 1509)		Jordan (n = 766)		Lebanon (n = 432)		Bahrain (n = 200)	
	Mean	SD		Mean	SD	Mean	SD	Mean	SD	Mean	SD	Mean	SD
**Age categories** (years)													
18–29 years	8.49	4.37	0.013*	8.72	4.42	8.50	4.44	8.33	4.25	7.44	3.98	8.07	3.93
30–49 years	8.11	5.02		6.60	3.76	8.71	5.27	7.70	4.89	7.15	4.99	8.00	3.92
50 years and above	8.25	4.81		6.17	6.24	7.97	5.31	13.00	5.89	8.89	2.47	8.16	3.53
**Gender**													
Males	8.22	4.53	0.004**	8.24	4.65	8.41	4.52	8.45	4.25	6.97	4.37	7.54	4.16
Females	8.59	4.35		8.94	4.27	8.57	4.60	8.18	4.42	7.60	3.93	8.11	3.87
**Marital status**													
Single	8.47	4.38	0.817	8.73	4.43	8.46	4.45	8.28	4.26	7.41	4.07	8.06	3.89
Married	8.41	4.70		8.10	4.19	8.76	4.93	8.13	4.89	7.86	3.91	7.65	4.24
Divorced	8.17	4.89		6.50	4.04	8.46	5.15	8.76	4.53	-	-	4.33	5.13
Widowed	9.31	6.65		14.50	2.12	8.08	6.50	11.50	10.61	-	-	-	-
**Field of study**													
Medical college	8.32	4.37	0.023*	8.81	4.40	8.35	4.56	8.10	4.24	7.21	4.03	7.99	3.92
Non-medical college	8.64	4.48		8.61	4.44	8.70	4.58	8.99	4.76	8.39	4.04	7.75	4.17
**Year level**													
First year	8.24	4.16	0.255	8.06	4.34	8.84	4.48	8.23	3.96	7.73	3.71	7.91	3.69
Second year	8.49	4.38		8.78	4.68	8.61	4.07	8.46	3.89	7.22	4.29	8.13	4.76
Third year	8.82	4.42		9.26	4.38	8.62	4.65	9.02	4.35	7.28	3.76	7.52	3.64
Fourth year	8.47	4.46		8.57	4.41	8.45	4.60	8.83	4.74	7.01	3.86	9.10	3.84
Fifth year	8.39	4.35		8.79	4.30	8.18	4.25	7.24	4.43	7.48	4.49	7.47	4.78
Sixth year	8.50	4.72		9.10	5.07	8.32	4.73	8.57	5.00	9.80	5.72	8.54	4.06
Higher education	8.28	4.71		8.16	4.41	8.77	5.23	7.77	5.03	7.83	4.24	7.58	3.81
**Income level**													
Less than 700$	8.51	4.40	0.299	8.73	4.41	8.48	4.49	8.30	4.34	7.45	4.17	8.01	3.86
700–1500$	8.34	4.23		8.32	4.47	8.80	4.29	8.23	4.36	7.55	3.63	8.03	3.90
1500–2100$	8.02	4.66		5.17	4.09	8.63	5.07	8.22	4.12	6.57	3.15	7.27	4.23
2100$ and above	8.31	4.74		8.36	4.57	8.36	4.86	8.25	5.13	7.89	4.29	8.21	4.17
**In general, do you suffer from an anxiety problem or use a treatment for anxiety?**													
No	7.94	4.28	< 0.001***	8.19	4.35	8.10	4.31	7.69	4.20	6.45	3.73	6.98	3.43
Yes	9.65	4.51		10.30	4.24	9.25	4.92	9.44	4.44	8.95	4.07	9.59	4.21
**Duration of mobile phone usage** (years):													
Less than one year	7.29	4.95	0.035*	7.34	5.27	8.13	5.94	7.00	2.78	7.00	5.66	4.75	4.99
1–5 years	8.36	4.40		8.62	4.44	8.27	4.47	8.02	4.23	7.25	4.14	8.28	4.55
6–10 years	8.46	4.32		8.74	4.38	8.44	4.46	8.34	4.20	7.50	3.81	8.40	3.86
10 years and above	8.57	4.52		8.78	4.39	8.68	4.69	8.49	4.69	7.41	4.55	7.60	3.76

*p < 0.05; **p < 0.01; ***p < 0.001

### Risk factors of mobile phone dependence

Binary logistic regression was used to identify risk factors of mobile phone dependence. Females were 15% at higher risk of developing mobile phone dependence compared to males (Odds ratio (OR): 1.15 (1.01–1.31), p ≤ 0.05). Having anxiety problem or using a treatment for anxiety was another important risk factor that increased the risk of developing mobile phone dependence by 75.0% (OR: 1.75 (1.54–1.99), p < 0.001), Table 5.

**Table 5:** Binary logistic regression analysis

Demographic variable	Odds ratio of being mobile phone dependent (95%CI)
**Age categories** (years)	
18–29 years (Reference group)	1.00
30–49 years	0.87 (0.67–1.14)
50 years and above	0.74 (0.42–1.31)
**Gender**	
Males (Reference group)	1.00
Females	1.15 (1.01–1.31)*
**Marital status**	
Single (Reference group)	1.00
Married	0.89 (0.71–1.11)
Divorced	1.30 (0.74–2.30)
Widowed	1.88 (0.68–5.19)
**Field of study**	
Medical college (Reference group)	1.00
Non-medical college	1.01 (0.89–1.14)
**Year level**	
First year (Reference group)	1.00
s year	1.02 (0.85–1.21)
Third year	1.11 (0.95–1.31)
Fourth year	0.95 (0.81–1.11)
Fifth year	1.06 (0.92–1.22)
Sixth year	1.00 (0.76–1.32)
Higher education	0.99 (0.81–1.21)
**Income level**	
Less than 700$ (Reference group)	1.00
700–1500$	1.08 (0.88–1.32)
1500–2100$	0.98 (0.72–1.33)
2100$ and above	0.82 (0.62–1.08)
**In general, do you suffer from an anxiety problem or use a treatment for anxiety?**	
No (Reference group)	1.00
Yes	1.75 (1.54–1.99)***
**Duration of mobile phone usage** (years):	
Less than one year (Reference group)	1.00
1–5 years	1.25 (0.49–3.24)
6–10 years	0.57 (0.28–1.17)
10 years and above	0.98 (0.50–1.94)

*p < 0.05; ***p < 0.001

## Discussion

Humans are social by nature, and interpersonal relationships are essential to their health. Living in a virtual world may have its implications at several aspects [29]. Numerous studies have documented the negative effects of excessive smartphone use on a variety of health factors, including sleep disturbance, anxiety, depression, changes in gene regulation, headaches, auditory and visual disturbances. exhaustion, memory loss, behavioral issues, and deficits in attention [30–40]. Therefore, the term nomophobia “no-mobile-phone phobia” was introduced to denote a psychological status where an individual develops a fear of being detached from his/her mobile phone connectivity [29]. Most studies reported that the prevalence is mainly among young adults. Therefore, this study aimed at assessing the prevalence of nomophobia in university students in five Middle Eastern countries.

In the current study, the highest mobile dependence score was observed in university students from Egypt, and the lowest mobile dependence score was observed in university students from Lebanon. Around one quarter (24.2%) the participating university students are diagnosed (symptomatically according to ICD-10 criteria for dependence syndrome) with mobile phone dependence. These results match those observed in three earlier studies, which found that the prevalence of mobile phone dependence (according to ICD-10 criteria) ranges between 18.5% and 39.6% [1, 12, 41]. Due to the large number of mobile phone users, this rate is notably high.

[26]Only one student in a research in the United States had an absent nomophobia score, whereas the bulk of the students (56.8%) scored in the moderate nomophobia range [42]. Nomophobia was found in 99.3% of Omani students, with the majority experiencing a moderate level of fear [43]. Students in Saudi Arabia had an average of 85.3% nomophobia, with 63.2% having mild nomophobia and 22.1% having severe nomophobia [44]. The frequency of moderate to severe nomophobia was roughly 93% in a research conducted in Bahrain [45]. Nomophobia was found in all of the participants in India, with roughly 82% suffering from severe nomophobia [46]. This rise in prevalence could be attributed by the fact that, especially during emergency such as the covid-19 pandemic, mobile phones have become a vital and distinctive technology for everyone communication [47–51].

In 2021, there were 5.2 billion unique users of smart phones, which represent an alarming 66.6% of global population. Out of this number 4.66 billion were internet user (representing 59.5% of global population), and 53.6% of the global population are active social media users (4.2 billion). In the middle east, internet users reached 95.7% (population 35.08 million), 99% (population 1.72 million), 78.2% (population 6.8 million), 57.3% (population 103.3 million), 66.8% (population 10.24 million) of the population, in Saudi Arabia, Bahrain, Lebanon, Egypt, and Jordan respectively. Whereas active social media users reached 79.3%, 87%, 64.3%, 47.4% and 61.5% of the population in Saudi Arabia, Bahrain, Lebanon, Egypt and Jordan respectively [52–55]. In comparison with the global figures, apart from Egypt, the rate of active social media users exceeded the global figures. Hence, the 24.2% of users suffering from nomophobia is alarming. Therefore, nomophobia needs to be a public health issue that worth collaboration. In the United States, 89% of adolescents aged 13 to 17 now own a smartphone [56]. Around 98.0% of the adult population have a mobile phone in the UK [57].

The most prevalent dependence criterion among our study population was impaired control, followed by withdrawal, and the least prevalent was harmful use. In a previous study of adolescents, inadequate control and withdrawal symptoms were identified as the most prevalent diagnostic criteria [12], which was in line with the literature [1]. Harmful use, on the other hand, was the least common [12]. These findings, however, contrast with those of a survey of resident doctors, which revealed that withdrawal was the most common criterion, followed by the neglect of alternative pleasures [1]. In comparison to adults, these data show that college students and young adults have poor self-control when it comes to managing mobile phone use and other activities.

Females were 15% at higher risk of developing mobile phone dependence compared to males (Odds ratio (OR): 1.15 (1.01–1.31), p ≤ 0.05). Methodological inconsistencies across studies made it difficult to reach a definitive conclusion regarding gender differences in nomophobia [58]. The findings of the current study largely agree with the results obtained by Nikhita and co-workers (2015), using the same tool, in India. A total of 31.33% of the sampled students (secondary school adolescents) demonstrated mobile phone dependence. Their results were not in agreement with that of our finding in terms of gender. Nikhita et al. findings revealed that nomophobia prevalence is significantly associated with gender (p = 0.003, OR = 1.91, CI: 1.23–2.99) with prevalence among male is higher than that of female. In our study the prevalence among female was higher [12]. This could be attributed to the tendency of females to overuse social media when compared to males [16]. On the other hand, a study in Australia (2020), reported that there was no association between gender and nomophobia [23]. Other studies also reported the lack correlation between nomophobia and gender [17, 59], while a study by Yildirim that targeted college students in Turkey found that females had higher levels of nomophobia [60]. Such mixed findings indicate gender differences may be affected by cultural difference among countries.

The findings of this study further revealed variations among countries in terms of the total mobile dependence scores suggesting the important role of cultural differences. Undeniably, the role of culture in affecting nomophobia has been studied, suggesting cultural perspective may substantially influence the technology behaviours [23, 61]. Yet, when the fear of being without a mobile phone produces such consequences as impaired control (57.6%), withdrawal symptoms (55.6%), decreased pleasure (49.4%), and intense desire (46.8%), there is a need to better understand and focus on nomophobia as a complex socio-technical phenomenon. Public health leaders need to address if higher levels of nomophobia translate into higher levels of risk for this vulnerable group, which might affect their capability to cope with prospective life challenges [21, 23, 59, 62].

In terms of age, we found that people between the ages of 18 and 29 had higher levels of mobile dependence; this finding is in line with the findings of a recent systematic review, which found that younger people are more prone to nomophobia [58]. Some explanations claim that this is due to the fact that young people are more familiar with contemporary technologies and practices than older ones [26].

The second risk factor that bring a lot of concern is pertinent to students having anxiety problems. Around one-third (29.9%) the study participants reported that they suffer from an anxiety problem or use a treatment for anxiety with an odd ratio of 1.75 (CI: 1.54–1.99, p < 0.001). Again, the percentage of students suffering from anxiety or taking medication for that is alarming. A previous meta-analysis in (2018) reported that there is a small-moderate association between mobile phone use and stress and anxiety (r = 0.22, p < 0.001, CI [0.17–0.28]). Therefore, a positive correlation was observed between the use of smart phone and the occurrence of stress and anxiety [63]. Such findings suggest that there is an urgent need to carry out further research into the level of anxiety caused using smart phones, its implications, and the measure to reduce that. It’s worth noting that the COVID-19 pandemic has had an impact on the mental health of several groups in society, especially university students [64]. All studies agreed that the epidemic has added a significant amount of stress to people’s lives, increasing their chances of acquiring anxiety, depression, and other mental diseases, particularly among university students [65–71].

Nomophobia is characterized by a number of psychological and physical symptoms induced by the unavailability of mobile phones. These symptoms include discomfort, stress, anxiety, anger, sleeplessness, and others [60]. A study suggested that nomophobia be included in the Diagnostic and Statistical Manual of Mental Disorders, fifth edition (DSM-V), noted that people suffering from pre-existing disorders such as anxiety, low self-esteem, and depression are more vulnerable to the effects of nomophobia [13]. These findings suggest that mental health disorders and nomophobia may have a bidirectional relationship. In the current study, approximately one-third (29.9%) of the study participants reported suffering from an anxiety problem or using a treatment for anxiety. Our analysis showed that having an anxiety problem or using a treatment for anxiety significantly increased the risk of developing mobile phone dependence by 75%. In confirmation of the current study’s findings, a Lebanese study conducted to evaluate the psychological conditions that could be connected to nomophobia demonstrated that a higher level of anxiety was significantly correlated with a higher nomophobia score [25]. Additionally, Veerapu et al., Sharma et al., and Ithnain et al. have also noted a positive correlation between anxiety and nomophobia in their studies [72–74]. Similarly, our survey showed that most of the students reported that they become annoyed when they are unable to find their mobile phone in the morning or were asked to minimize their mobile phone use.

Concerning the amount of daily time spent using mobile phones in our study, the mean estimated time in the present study was 186.4 (94.4) minutes. In a study conducted in Saudi Arabia among university students, 61% reported spending at least five hours per day using their mobile phones [75]. In India, Bartwal et al. reported that 62.1% of the medical students spend three or more hours using their mobile phones, while Gupta et al. reported that only 17.8% of the students were spending more than three hours on their mobile phone [41, 76]. Nomophobia has been linked to the amount of time spent on mobile phones in previous studies; Pavithra et al. and Sahin et al. found that nomophobia was associated with increased daily mobile phone use [77, 78]. Additionally, Daei et al. found that the frequency of mobile phone use can be a predictor of nomophobia [79].

The present findings highlight the adverse impact of nomophobia on undergraduate students and emphasize the need to support individuals or students suspected of having nomophobia [80]. Nomophobia could develop into a substantial public health concern in the coming years if it were not detected and addressed promptly. As proven by the literature, the effect of nomophobia on the students’ mental and physical health may extend to their academic performance, dedication to learning, and their ability to build and maintain relationships [81–83]. To prevent these negative consequences and encourage safe and effective mobile phone usage among this population, it is crucial to raise the students’ awareness of this growing problem and encourage them to engage in alternative activities during their free time to minimize the use of mobile phones. Early screening of anxiety symptoms and nomophobia, especially in undergraduate females using standardized tools, is recommended. Future research should focus on providing effective solutions and clear guidelines for managing nomophobia.

Nevertheless, living in the modern world without a smart phone is not negotiable and no one can deny the added value of smart phones. However, policy makers need to identify means to shift the undergraduates students from personal relationship with their mobiles towards their dynamic offline reality rather than encouraging more use of the smart phones [23]. Raising awareness is required by policy makers, parents and educators. This might be accomplished through campaigns that discuss positive and negative behaviors pertinent to smart phone use.

This is the first large study to assess the prevalence levels of nomophobia among five Middle Eastern countries. This study investigated the relationship between smart phones use pattern and mobile dependence severity which will allow a better understanding of how an extreme dependence and connection to mobile phones may result in nomophobia at various levels. However, this study had few limitations. First, the use of an online self-reported data is subject to bias. Some of the participants may have overestimated or underestimated their responses to the questionnaire, impacting the accuracy of findings. Second, as it was an online questionnaire that utilised convenient sampling technique, we were unable to obtain response rate and the study sample might not be generalizable to the whole study population. Finally, the results from the present study are centred on a convenient sample and may cause volunteer bias (i.e., individuals who approved to participate in the online survey may be more concerned in smartphones) and who were mostly engaged through social media platforms and therefore may not be an actual representative of the general populations. Therefore, our findings should be interpreted carefully.

## Conclusion

Mobile phone dependence is common among university students in Arab countries in the Middle East region. Females and those with pre-existed anxiety disorders were more likely to develop mobile phone dependence. Future studies exploring useful interventions to decrease mobile phone dependence are warranted.

## Data Availability

The datasets supporting the conclusions of this study are available from the corresponding author upon request.

## Abbreviations

MPPU: Mobile phone problematic use
PCPU: problem cell phone use
MPD: mobile phone Dependence
DSM-IV: Diagnostic and Statistical Manual of Mental Disorders
SPSS: Statistical package for social sciences
CI: Confidence interval
SMS: short-message-service
